# Metabolomic Profiling of 13 Diatom Cultures and Their Adaptation to Nitrate-Limited Growth Conditions

**DOI:** 10.1371/journal.pone.0138965

**Published:** 2015-10-06

**Authors:** Mariusz A. Bromke, Jamal S. Sabir, Fahad A. Alfassi, Nahid H. Hajarah, Saleh A. Kabli, Abdulrahman L. Al-Malki, Matt P. Ashworth, Michaël Méret, Robert K. Jansen, Lothar Willmitzer

**Affiliations:** 1 Max Planck Institute of Molecular Plant Physiology, Am Mühlenberg 1, 14476, Potsdam, Germany; 2 University of Texas at Austin, Department of Integrative Biology, University of Texas at Austin, Austin, Texas, 78712, United States of America; 3 Biotechnology Research Group, Department of Biological Sciences, Faculty of Science, King Abdulaziz University, Jeddah, 21589, Saudi Arabia; Stazione Zoologica Anton Dohrn, Naples, ITALY

## Abstract

Diatoms are very efficient in their use of available nutrients. Changes in nutrient availability influence the metabolism and the composition of the cell constituents. Since diatoms are valuable candidates to search for oil producing algae, measurements of diatom-produced compounds can be very useful for biotechnology. In order to explore the diversity of lipophilic compounds produced by diatoms, we describe the results from an analysis of 13 diatom strains. With the help of a lipidomics platform, which combines an UPLC separation with a high resolution/high mass accuracy mass spectrometer, we were able to measure and annotate 142 lipid species. Out of these, 32 were present in all 13 cultures. The annotated lipid features belong to six classes of glycerolipids. The data obtained from the measurements were used to create lipidomic profiles. The metabolomic overview of analysed cultures is amended by the measurement of 96 polar compounds. To further increase the lipid diversity and gain insight into metabolomic adaptation to nitrogen limitation, diatoms were cultured in media with high and low concentrations of nitrate. The growth in nitrogen-deplete or nitrogen-replete conditions affects metabolite accumulation but has no major influence on the species-specific metabolomic profile. Thus, the genetic component is stronger in determining metabolic patterns than nitrogen levels. Therefore, lipid profiling is powerful enough to be used as a molecular fingerprint for diatom cultures. Furthermore, an increase of triacylglycerol (TAG) accumulation was observed in low nitrogen samples, although this trend was not consistent across all 13 diatom strains. Overall, our results expand the current understanding of metabolomics diversity in diatoms and confirm their potential value for producing lipids for either bioenergy or as feed stock.

## Introduction

Diatoms are eukaryotic phototrophs and are able to very efficiently sequester water-dissolved carbon dioxide. It is estimated that diatoms are responsible for up to 20% of the global primary production [1,2]. Diatoms can also efficiently use other available nutrients such as silica (main elemental component of their cell wall) and nitrogen to maintain their cell cycle [3–6]. Like in other organisms, the metabolism of carbon and nitrogen is closely coupled in diatoms. Limitation of one of these elements leads to severe changes in fluxes and utilization of the other [7].

Diatoms are very efficient at nutrient utilization and thus successful organisms in various environments [8] but can be readily adapted into prolific cultures; in these artificial conditions they often outcompete other algae in mixed cultures [9,10]. They are also relatively resistant to various pathogens [11,12]. Hence, they are highly regarded for their potential in producing feed or as a sustainable source of valuable substances, especially biofuels. Producing renewable, sustainable and carbon neutral fuels are of paramount importance to security and economic prosperity. One way of obtaining desired production is to manipulate culturing conditions such as nutrient availability. A combination of nitrogen and silicon limitation might be used to induce accumulation of lipids up to 50% of dry weight [13]. The genetic transformation of diatoms was initiated with the aim of improving oil production [14]. This approach might be very promising especially with the development of genomics tools for diatoms [15–19]. Moreover, marine algae synthesize diverse lipids, such as phosphatidylcholine, which might be important for food or feed production [20].

There are estimated 100,000 extant species of diatoms colonizing various environments [21]. With such diversity, we expect diatoms to produce a wide range of products depending on their local environment and evolutionary history. We already know diatoms produce several marketable natural products—biogenic silica and lipids—but as yet the yields have not been as cost-efficient for mass production. By bioprospecting for lipids and additional products from diatoms and other marine algae, we can increase the efficiency of farming these algae with the extraction of multiple products from cultures as well as discover novel productsfor future biotechnological applications [22,23]. To gain insight into the metabolism of diatoms we have selected cultures from a widest systematic range of diatom lineages ever sampled: *Amphitetras antediluviana*, *Biddulphia biddulphiana*, *Cerataulina daemon*, *Chaetoceros simplex*, *Eunotogramma sp*., *Hemiaulus sinensis*, *Leptocylindrus danicus*, *Rhizosolenia setigera*, *Thalassionema frauenfeldii*, *Thalassiosira pseudonana* (two strains CCMP1007 and CCMP1335) and *Thalassiosira weissflogii* (two strains CCMP1587 and CCMP1336) and studied their metabolome under nitrogen-rich and nitrogen-limiting conditions. To fully control the growth medium and nutrient levels diatoms were cultivated in artificial sea water in standard f/2 medium [24] and low-nitrate containing media. We have studied the adaptation to the applied conditions by measuring levels of 142 glycerolipids and changes in content of 96 polar compounds (mostly representing primary metabolites). Here we compare different N-limitation adaptation responses in 13 diatom cultures and describe the diversity of identified metabolites.

## Materials and Methods

### Algal cultures

Diatom starter cultures were obtained from National Center for Marine Algae and Microbiota in Bigelow, USA (*Thalassiosira pseudonana* CCMP1335, *T*. *pseudonana* CCMP1007 and *T*. *weissflogii* CCMP1587, *T*. *weissflogii* CCMP1336, *Chaetoceros simplex* CCMP200) and from a collection at University of Texas at Austin (*Amphitetras antediluviana*, ECT3627; *Biddulphia biddulphiana*, ECT3902; *Cerataulina daemon*, AP8; *Eunotogramma sp*. *(laeve)*, AP8Eunoto; *Hemiaulus sinensis*, 24I10-1AHemi; *Leptocylindrus danicus*, ECT3929araphid3; *Rhizosolenia setigera*, 25VI12-2ARhizo; *Thalassionema frauenfeldii*, ECT3929ThalXL). The cultures of diatoms were maintained in f/2 medium [24]. To control the chemical composition of the growth conditions, the medium was prepared by dissolving the f/2 salts and vitamins in artificial sea water, which was prepared from inorganic salts according to recipe for ESAW by Berges et al. [25]. The light intensity was 80 μmol/m^2^/s and the temperature was kept at 22°C throughout the 16 h day/8 h night regime. In order to induce nitrogen limitation and maintain high cell yield per flask, an inoculum from a stationary culture was transferred into a fresh full nitrogen f/2 medium (880 μM NO_3_^-^) and to a medium containing 20% of the nitrate in f/2 concentration. Each culture was represented by five replicates in these two nutrition conditions.

To ensure induction of the nitrogen limitation response, diatom cultures were harvested two days after the concentration of nitrate in low-nitrogen medium fell below the limit of detection. Diatoms were harvested by filtration onto a glass microfibers filter GF/C (Whatman) and frozen in liquid nitrogen. Usually 40 ml were harvested, with the exception of dense cultures of *Thalassiosira* in which 15–20 ml were used. The harvested samples of diatoms were kept in -80°C prior to the metabolite analyses. Cell number in each flask at the point of harvest was estimated using a Neubauers’ haemocytometer. In case of *Thalassiosira* cultures cell density was measured with Coulter Counter Z2 (Beckman).

The nitrate levels in media were estimated using an AQUANAL-plus nitrate test kit (Sigma-Aldrich). To increase reproducibility the original protocol was adapted as follows. The kit reagents A, C, and D were dissolved in distilled water: 1 spoon per 1 ml. A 1 ml sample of the medium was spun at 21,000 *g* for 2 min to remove cells and debris. In the following step, 800 μl of the supernatant was mixed with 200 μl of the dissolved reagent A and 26 μl of the reagent B. After vortexing, 200 μl of each reagent C and D were added. The reaction tube was vortexed and left for 5 min before spectrophotometric (Ultrospec2000, Pharmacia Biotech) measurement at 530 nm wavelength. The artificial sea water f/2 medium without added nitrate was used as the blank sample. The lower detection level of nitrate in the medium used in these experiments was 2.5 μM.

### Analytics and data analysis

Metabolite extraction from diatoms was performed using the method of Giavalisco et al. [26] and Bromke et al. [7]. In short, cells collected on filters were extracted with a cold mixture of methyl-*tert*-butyl-ether:methanol (3:1), spiked with 0.1 μg/ml phosphatidylethanolamine (PE 34:0 [17:0/17:0]), phosphatidylcholine PC 34:0 [17:0/17:0]) and 13C-sorbitol as internal standards. To facilitate cell disruption, samples were shaken and incubated in a cooled sonic bath for 10 min. The subsequent addition of a water:methanol (3:1) mixture to the extract resulted in the formation of two liquid phases (polar and non-polar phase). The lipid containing non-polar phase was aspirated, dried in vacuum and kept in –20°C prior to the profiling. The fractionation of lipids was performed by a Waters Acquity UPLC system using a C_8_ reversed-phase column (100 mm × 2.1 mm × 1.7 μm particles; Waters). For the UPLC gradient the mobile phases consisted of two solvents: solvent A, 1% 1 M NH4Ac and 0.1% acetic acid in water; and solvent B, acetonitrile/isopropanol (7:3, 1% 1 M NH4Ac, 0.1% acetic acid), with an injection volume of 2 μl. The following gradient profile was applied: 1 min, 55% B; 3 min, linear gradient from 55% B to 75% B; 8 min, linear gradient from 75% B to 89% B; 3 min, linear gradient from 89% B to 100% B. After washing the column for 4.5 min with 100% B, the mixture was set back to 55% B and the column was re-equilibrated for 4.5 min (24.5 min total run time), with a flow rate of the mobile phase of 400 μl/min. The mass spectra were acquired using an Orbitrap mass spectrometer (Exactive, Thermo Scientific). The spectra were recorded alternating between full-scan and all-ion fragmentation-scan modes, covering a mass range from 100 to 1500 *m*/*z* [26]. Chromatograms from the UPLC-MS analysis were analysed and processed with REFINER MS^®^ 7.5 (GeneData). The software extracted analytical features (analytes described by their mass, retention time, and associated intensity) from raw files generated by the mass spectrometer. Further processing of the data included the removal of the fragmentation information and chemical noise (constantly eluting non-biological compounds derived from the column or the employed UPLC solvents) as well as the retention time alignment. The output data were normalized to internal standard PC 34:0 due to lower number of co-eluting analytes. Peaks were annotated with help of an in-house database of lipid compounds. Further processing was performed with scripts written in R [27], which filtered aligned data matrices to keep annotated peaks and 1200 analytes (a number based on previous experience with analysis of lipidomic data) of highest intensity. Additionally, analytes that showed up in less than four replicates were removed. The data were normalized to the cell density at the time of harvest, scaled to the median of the data matrix and log2-transformed. The data are available in the S1 Table. Visualization of lipid data was performed with R. The generation of Primary Component Analysis plots used the ‘pca’ function with the default "ppca" method. Heatmaps were generated using function ‘heatmap.2’ (a part of a ‘gplots’ package), which utilizes Euclidean distance as a basis for hierarchical clustering.

The polar fractions of the metabolite extracts were derivatizatized and prepared for the GC-MS-based analysis. Shortly thereafter, the dried extracts were resolved in a mixture of methoxyamine hydrochloride in pyrydine and derivatized with N-methyl-N-(trimethylsilyl)-trifluoroacetamide (MSTFA). The samples were analyzed, coupled to an Agilent 6890 gas chromatographer coupled with a time-of-flight mass spectrometer (Agilent Technologies). Details of the method are described by Lisec et al. [28]. Chromatograms were evaluated in Leco ChromaTof (Agilent technologies) software and exported in.cdf format. Further processing of chromatograms (peak detection, retention time alignment and library matching) was performed using the TargetSearch R package from bioconductor [29]. To offset errors introduced through sample-handling the intensity of each analyte peak was divided by the intensity of ^13^C-sorbitol (the polar-phase internal standard). The data were normalized to the cell density of every culture. The statistical significance of changes between N-replete and N-limiting conditions was tested with the T-test.

Prior to further analysis and visualization the metabolomic data were log2-transformed. The data are available in S2 Table. The data visualization with the heatmap was performed as described above. Changes in p-values less than 0.05 were marked with asterisks.

## Results

Different species and isolates of diatoms were acquired from algal collections and grown under laboratory conditions. Cultures were selected on the basis of their culturing properties (ease of handling, low or no adhesion to the glass surfaces) and ability to grow fast at a relatively high temperature of 22°C. To induce changes in lipid metabolism of diatoms, they were cultivated in standard f/2 medium [24] as well as in this medium containing only 20% of the f/2-medium’s nitrate (176 μM). Samples of each culture (full nitrate and low nitrate containing media) were grown for the same time and harvested when nitrate was depleted in the low-nitrate medium. Until the time point at which nitrate was depleted, no major changes in growth rate were observed between the full f/2 and the low nitrate cultures. For each condition five biological replicates were independently grown, harvested and analysed. By application of polar and non-polar solvent mixtures for extraction of each sample we obtained two phases of extracts that were analysed separately with use of gas and liquid chromatography, respectively. The data collected in both approaches were used to describe metabolism of the diatom cultures in nitrogen-replete and nitrogen-limited conditions.

### Growth in nitrogen-deplete or nitrogen-replete conditions affects lipid accumulation but has no major influence on lipid patterns

**Fig 1:**
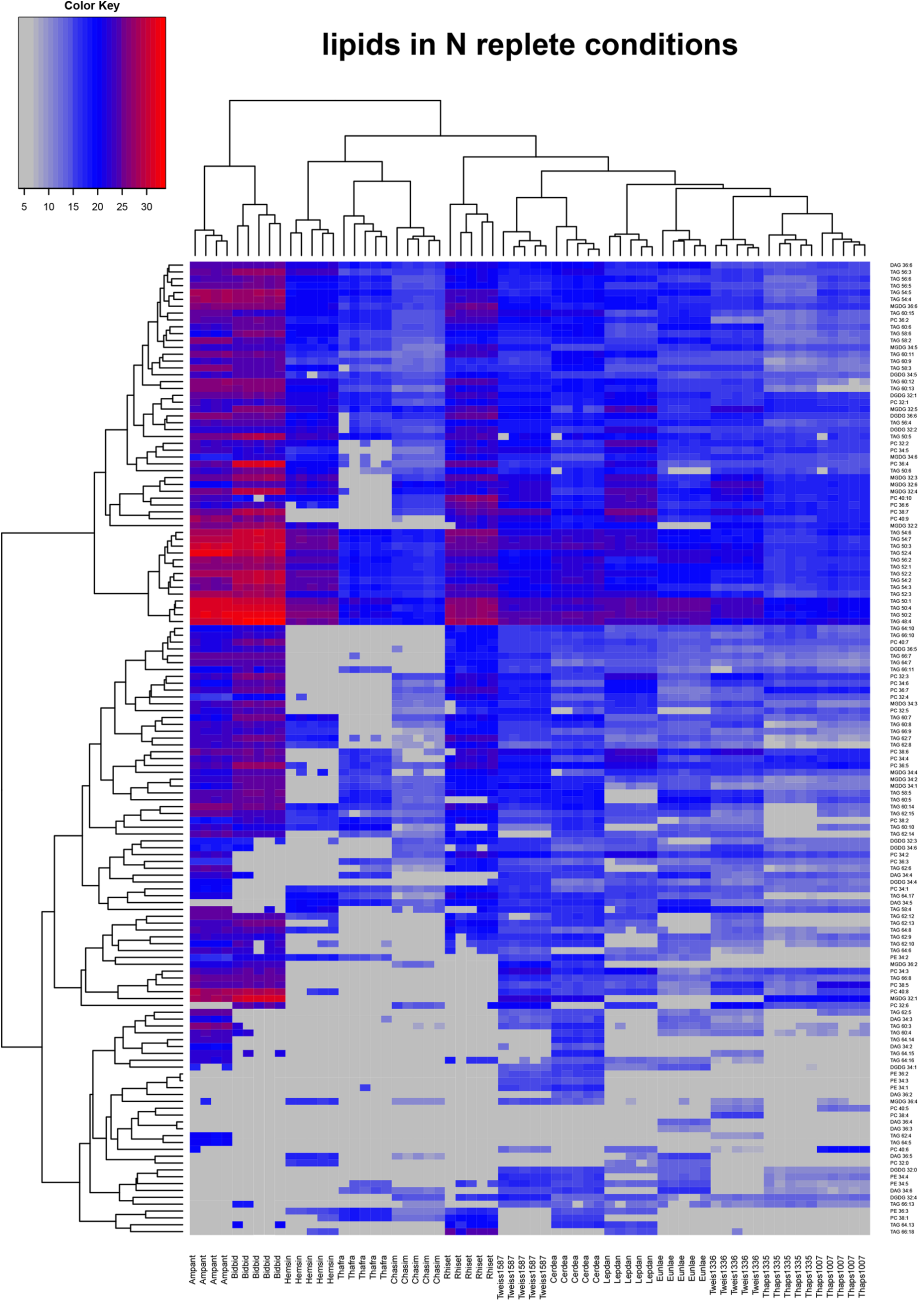
Heatmap of 142 lipid analytes from the nitrogen-replete cultures of diatoms. A blue-red scale of colours represents low-to-high relative content of any given analyte. The data were clustered on basis of the Euclidean distance. Analytes not detected are marked gray. Following abbreviations were used: Ampant, *Amphitetras antediluviana*; Bidbid, *Biddulphia biddulphiana*; Cerdea, *Cerataulina daemon*; Chasim, *Chaetoceros simplex*; Eunlae, *Eunotogramma sp*.; Hemsin, *Hemiaulus sinensis*; Lepdan, *Leptocylindrus danicus*; Rhiset, *Rhizosolenia setigera*; Thafra, *Thalassionema frauenfeldii*; Tp1007, *Thalassiosira pseudonana* CCMP1007; Tp1335, *Thalassiosira pseudonana* CCMP 1335; Tw1587, *Thalassiosira weissflogii* CCMP 1587; Tweiss1336, *Thalassiosira weissflogii*.

**Fig 2:**
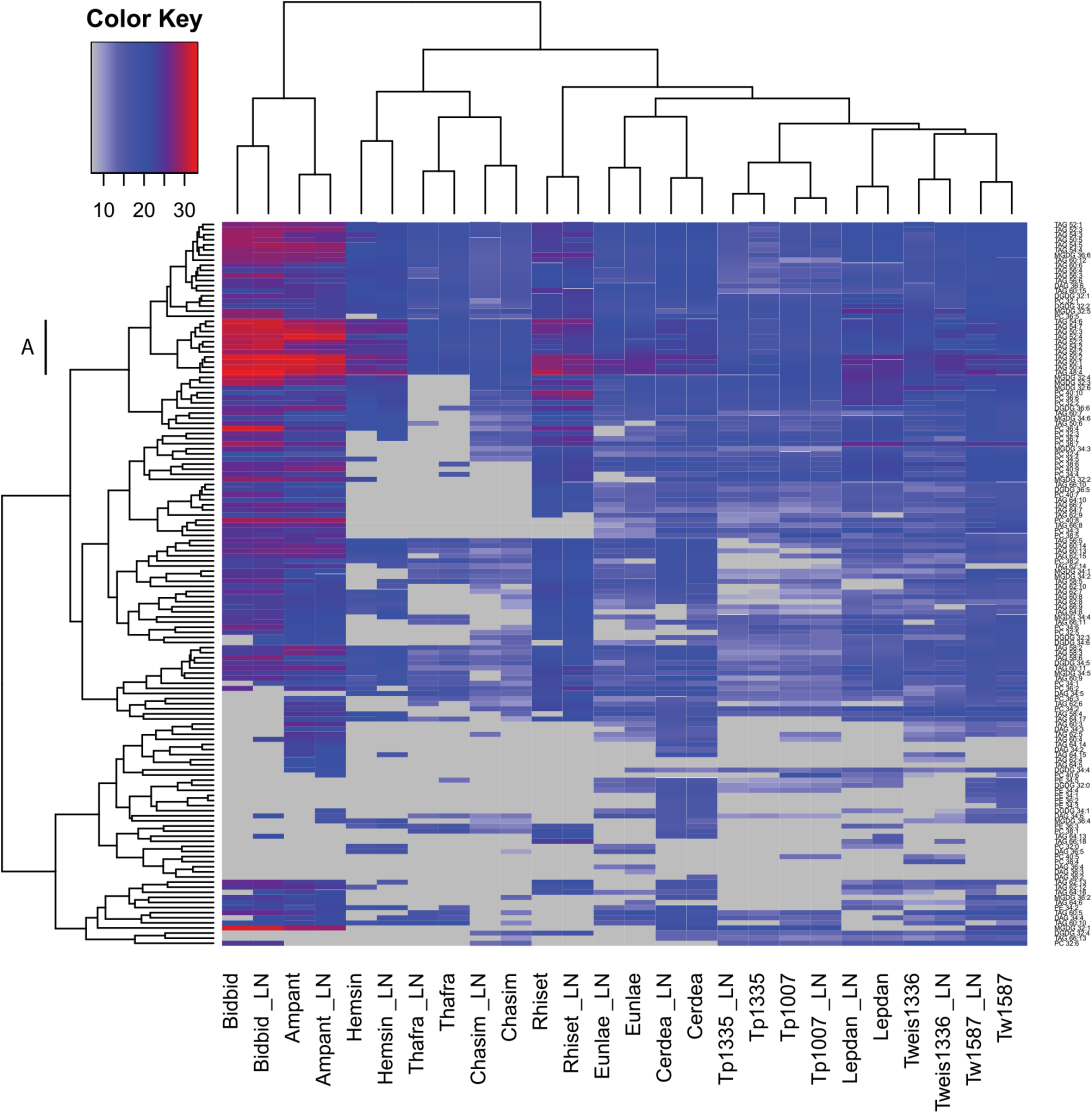
Heatmap of 142 annotated lipids measured in nitrogen-replete and nitrogen-depleted cultures of diatoms. A blue-red scale of colours represents low-to-high relative content of any given analyte. The data were clustered on basis of the Euclidean distance. Analytes not detected are marked gray. Following culture abbreviations were used: Ampant, *Amphitetras antediluviana*; Bidbid, *Biddulphia biddulphiana*; Cerdea, *Cerataulina daemon*; Chasim, *Chaetoceros simplex*; Eunlae, *Eunotogramma sp*.; Hemsin, *Hemiaulus sinensis*; Lepdan, *Leptocylindrus danicus*; Rhiset, *Rhizosolenia setigera*; Thafra, *Thalassionema frauenfeldii*; Tp1007, *Thalassiosira pseudonana* CCMP1007; Tp1335, *Thalassiosira pseudonana* CCMP 1335; Tw1587, *Thalassiosira weissflogii* CCMP 1587; Tweiss1336, *Thalassiosira weissflogii*. The “LN” suffix marks samples from low nitrogen growth conditions.

From the data obtained in the chromatographic analysis of the non-polar phase we selected almost 1200 high-quality features. Out of this number we were able to annotate and determine relative levels of 142 peaks representing lipid species in extracts from 13 diatom cultures (Fig 1). These lipids belong to six classes of glycerolipids: diacylglycerols (DAG; 10 analytes), phosphatidylethanolamines (PE; 7 analytes), phosphatidylcholine (PC; 31 analytes), monogalactosyldiacylglycerols (MGDG; 15 analytes), digalactosyldiacylglycerols (DGDG; 11 analytes), and triacylglycerols (TAG; 68 analytes). There were differences between analysed cultures in number of detected analytes. We measured as many as 129 and 123 analytes in nitrogen-replete and depleted cultures of *Cerataulina daemon*, respectively (Figs 1 and 2). On the other hand, we were able to annotate as few as 64 and 60 lipid species in *Thalassionema frauenfeldii* cultivated in high- and low-nitrogen, respectively (Figs 1 and 2). There were 32 lipid species common to all samples and out of this number, 25 represented TAG of different total carbon atoms in their acyl chains, ranging from 48 to 66 C atoms.

One of the main aspects of our study was to assess the influence of different culturing conditions (low versus high availability of nitrate) on lipid composition of diatoms. As shown in Figs 1 and 2, different amounts of nitrate available changed the lipid profiles leading to distinct patterns; however the species remained the most important discriminating factor. This means that the lipid analytes diversity in low nitrate cultures resembled the profile of the control, the high nitrate medium.

Whereas specific lipid composition was only slightly influenced by the nitrate availability, a change in lipid concentration was observed. Fig 2 shows a comparative analysis of the lipid profiles obtained from diatoms grown under low and high nitrate conditions in growth media. In agreement with other data [30,31], an increase in lipid species mostly of the TAG type was recognizable. However, this was not true for all diatom species analysed. The accumulation of TAG was especially visible in dense cultures of *Thalassiosira weissflogii* and *Thalassiosira pseudonana*. Surprisingly there was no specific pattern of changes in content of nitrogen-containing phospholipids, PC and PE. Also other classes of lipids showed no specific changes induced by the low-nitrogen in the growth medium.

The most abundant group of lipids were TAG with 50 carbon atoms in their acyl chains. The intra-lipid species correlation for these TAG (as well as TAG 48:X and TAG 52:X) formed a high-content block across the heatmap (Fig 2, cluster A). The cluster A contains 11 TAG species: TAG 48:4, TAG 50:1; TAG 50:2, TAG 50:3, TAG 50:4, TAG 52:2, TAG 52:4, TAG 54:2, TAG 54:6, TAG 54:7, TAG 56:2. The richest sources of these lipids were cultures of *B*. *biddulphiana*, *A*. *antediluviana*, and *R*. *setigera*. In most samples we detected TAGs of a high mass, which probably represent triacylglycerols with three poly-unsaturated fatty acids. We detected and annotated TAG 66:18, which was observed only in *L*. *danicus*, *T*. *weissflogii* CCMP1336, and *R*. *setigera*, the latter species was especially rich in this compound. There was also a high abundance of TAG 60:15 in lipid extracts.

Generally, our measurements showed that *A*. *antediluviana* and *B*. *biddulphiana* had the highest content of annotated lipids per cell but lowest cell density. The lowest amounts of lipids were observed in *Ch*. *simplex* and *T*. *frauenfeldii* extracts. Annotated lipid data are in S2 Table.

### PCA allows clear discrimination of diatom species

**Fig 3:**
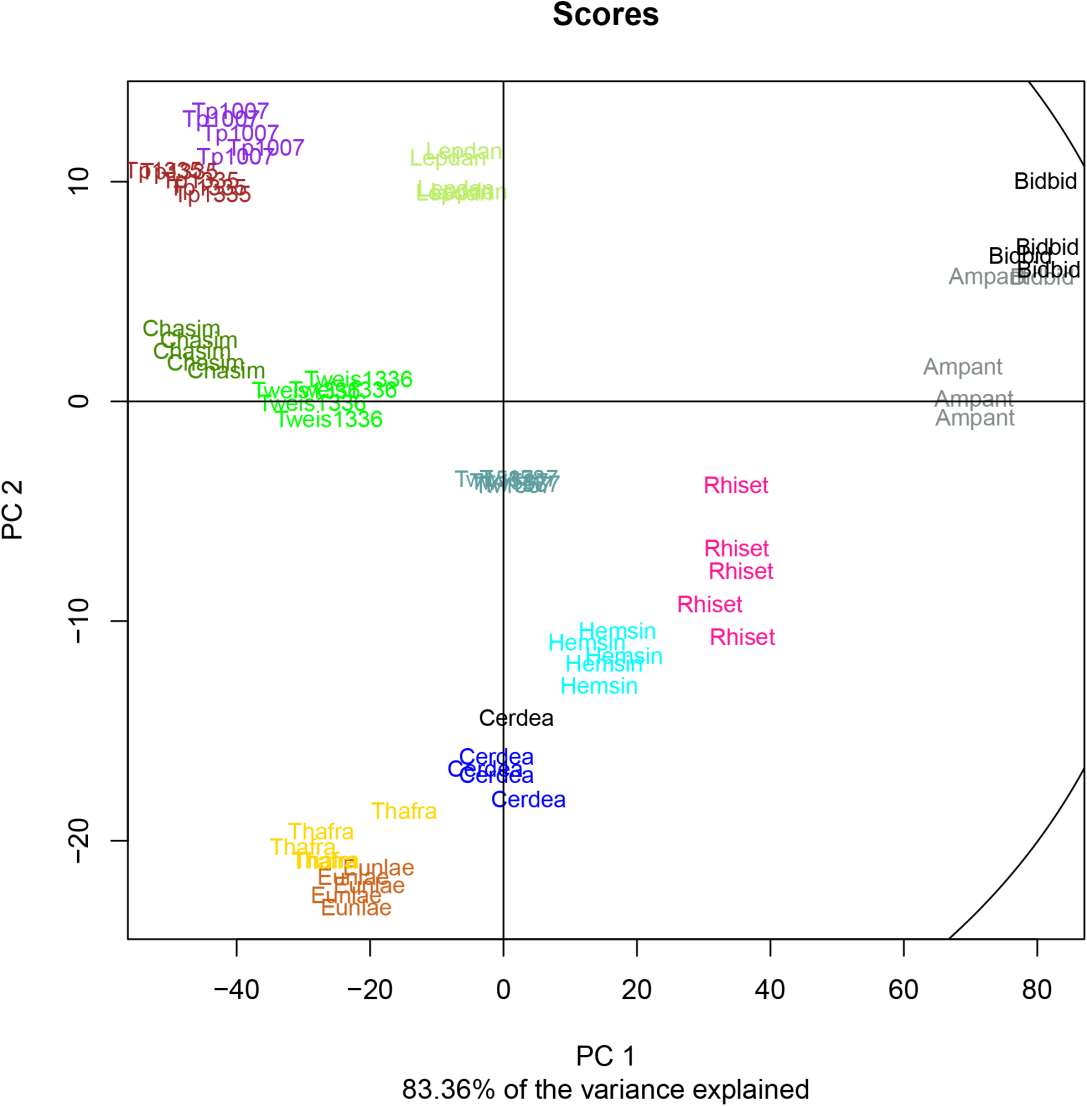
Principal Component Analysis of the lipid profiles of 13 analysed cultures grown in nitrogen-replete conditions based on 142 annotated lipid analytes (32 analytes common for all samples). Each replicate is represented by a coloured abbreviation of the culture name: Ampant, *Amphitetras antediluviana*; Bidbid, *Biddulphia biddulphiana*; Cerdea, *Cerataulina daemon*; Chasim, *Chaetoceros simplex*; Eunlae, *Eunotogramma sp*.; Hemsin, *Hemiaulus sinensis*; Lepdan, *Leptocylindrus danicus*; Rhiset, *Rhizosolenia setigera*; Thafra, *Thalassionema frauenfeldii*; Tp1007, *Thalassiosira pseudonana* CCMP1007; Tp1335, *Thalassiosira pseudonana* CCMP 1335; Tw1587, *Thalassiosira weissflogii* CCMP 1587; Tweiss1336, *Thalassiosira weissflogii*.

In order to obtain further insight into the diversity of the diatom lipids, the data were subjected to a principal component analysis (PCA, Fig 3). Two main conclusions were apparent: first, all 13 diatom strains could be separated from each other in a non-supervised fashion, clearly proving the diversity and species specificity of the lipid profiles; second, all five biological replicates of each species clustered together supporting the statistical relevance of our findings and the reproducibility of the culture conditions. The analysed data set contained profiles of 142 annotated analytes. The first two principal components accounted for more than 83% of the variance (Fig 3). The compound with the strongest influence on resolution was PC 40:6, being on extremes of principal component 1 and 2. This lipid compound was detected only in extracts of *T*. *pseudonana* CCMP1007, *T*. *weissflogii* CCMP1336, *T*. *weissflogii* CCMP1587, and *Leptocylindrus danicus*. All *T*. *pseudonana* profiles clustered very closely, and the two isolates CCMP1335 and CCMP1007 of *T*. *pseudonana* were not well discriminated. This was also evident in the heatmap (Fig 1), where we used a colour scale for visualization of individual data points in lipid profiles. The hierarchical clustering analysis applied to profiles of the other pair of related isolates of *T*. *weissflogii* showed a surprising difference. In consequence, the isolate *T*. *weissflogii* CCMP1336 seemed to be more similar to *T*. *pseudonana* profiles than to the *T*. *weissflogii* CCMP1587, which clustered together with *C*. *daemon*.

### Primary metabolites

**Fig 4:**
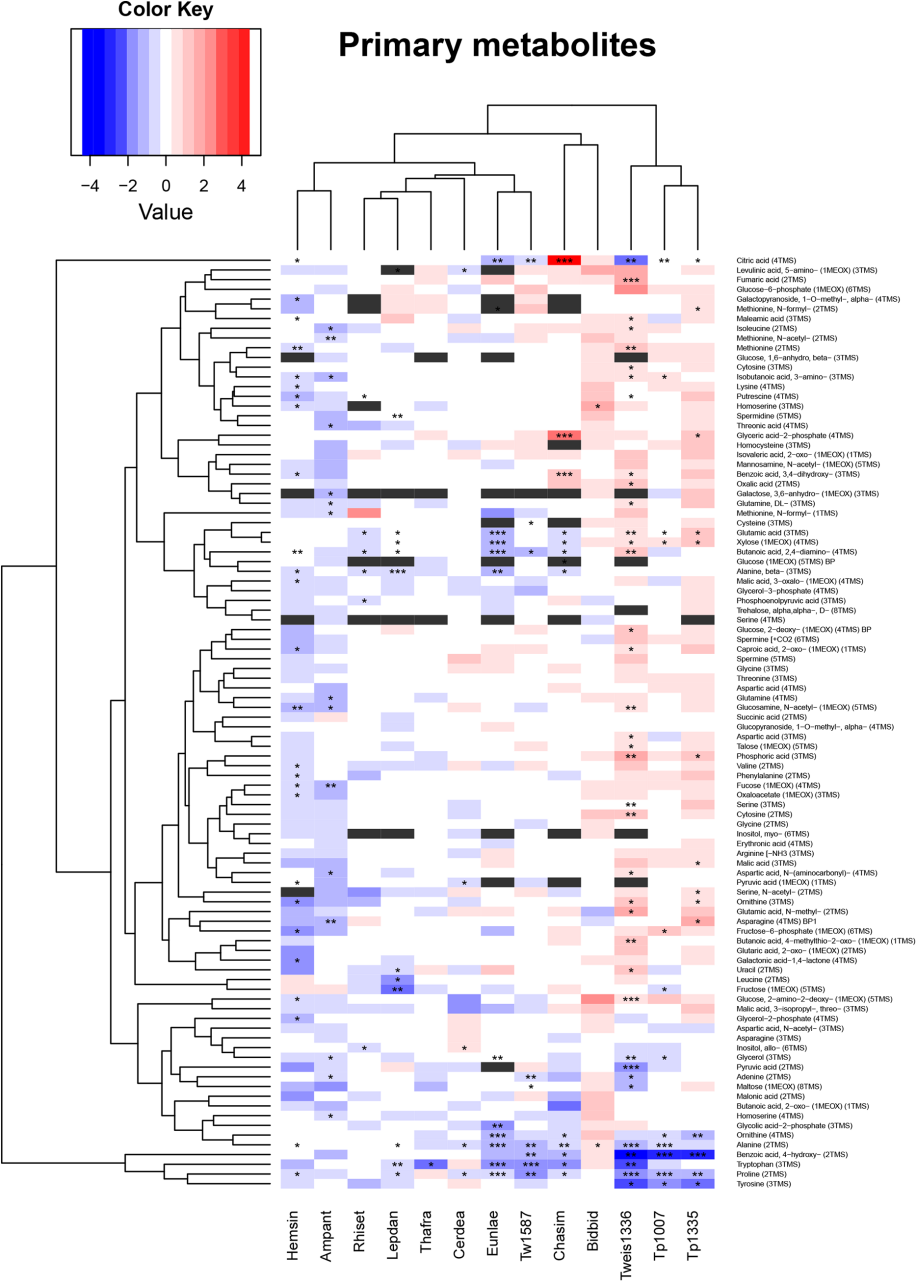
Heatmap of 96 primary metabolite changes in nitrogen-depleted cultures of diatoms. A blue-white-red scale of colours represents none-to-high relative content of a polar analyte. Asterisks mark Anova p-values below 0.05 (‘*’ < 0.05; ‘**’ < 0.01; ‘***’ < 0.001). The data were clustered on basis of the Euclidean distance. Analytes not detected are marked gray. The histogram presents the density of data points. Following culture abbreviations were used: Ampant, *Amphitetras antediluviana*; Bidbid, *Biddulphia biddulphiana*; Cerdea, *Cerataulina daemon*; Chasim, *Chaetoceros simplex*; Eunlae, *Eunotogramma sp*.; Hemsin, *Hemiaulus sinensis*; Lepdan, *Leptocylindrus danicus*; Rhiset, *Rhizosolenia setigera*; Thafra, *Thalassionema frauenfeldii*; Tp1007, *Thalassiosira pseudonana* CCMP1007; Tp1335, *Thalassiosira pseudonana* CCMP 1335; Tw1587, *Thalassiosira weissflogii* CCMP 1587; Tweiss1336, *Thalassiosira weissflogii*. The “LN” suffix marks samples from low nitrogen growth conditions.

The polar phase of each extract was used to measure low molecular size compounds representing mostly primary metabolites. The extracts were chemically derivatized and analysed by CG-MS. Obtained spectra were compared with the Golm Metabolomics Database (gmd.mpimp-golm.mpg.de). This resulted in a list of 96 polar analytes representing primary metabolites (Fig 4). Due to the nature of the derivatization process some metabolites were represented by two analytes, representing different derivatization products of the same metabolite. The largest changes observed in the data set were accumulation of citric acid (more than 28 fold) in cells of *Ch*. *simplex* (slight accumulation was also observed in *H*. *sinensis*, *B*. *biddulphiana*, and *T*. *pseudonana* CCMP1335) and reduction of 4−hydroxy−benzoic acid levels in three of four *Thalassiosira* species (8 to 20 fold less than in controls). These three diatom cultures (*T*. *pseudonana* CCMP1335, *T*. *pseudonana* CCMP1007, *T*. *weissflogii* CCMP 1336) also exhibited a distinctive accumulation of many metabolites during the treatment. Even more metabolites showed elevated levels in cells of *B*. *biddulphiana*. In contrast, *A*. *antediluviana* and *H*. *sinensis* showed reduced levels for most of analytes measured. The remaining diatom cultures in this experiment showed mixed phenotypes, in which levels of some analytes were increased and for some these levels dropped in the low nitrogen conditions.

The hierarchical clustering of metabolite changes showed a distinctive cluster of metabolites with reduced levels (bottom of the Fig 4). This was not uniform across the studied cultures, though none of the increased levels could be confirmed by the t-test. This cluster contained 4 nitrogen-containing metabolites: tryptophan, tyrosine, proline and 4-hydroxy-benzoic acid. Also, alanine and ornithine levels were reduced in most of the analysed cultures. The exception to this pattern was *B*. *biddulphiana*, which seemed to accumulate alanine and homoserine. Many other analytes showed elevated levels in cells of nitrogen-limited *B*. *biddulphiana*, but the p-value calculated was above the 0.05 threshold.

Contrasting responses to the N-limitation were observed in the case of xylose, glutamic acid and 2,4−diaminobutanoic acid (Fig 4). These three metabolites were found in reduced levels in cells of *L*. *danicus*, *Eunotogramma sp*. and *Ch*. *simplex*. Also other diatoms showed reduced levels of one or two of the metabolites. On the other hand three *Thalassiosira* cultures (*T*. *pseudonana* CCMP1335, *T*. *pseudonana* CCMP1007, *T*. *weissflogii* CCMP 1336) were accumulating these compounds during the experiment.

## Discussion

### Lipid profiling

One of the conclusions from these experiments is that lipid profiling is powerful enough to be used as a molecular fingerprint to characterize a wide phylogenetic diversity of diatom species. This was shown with the Pearson’s correlation-based clustering of profile data and displayed on the heatmap (Fig 1). This observation is also supported by the PCA (Fig 3), where clustering of biological replicates confirms the reproducibility of measurements and specificity of lipidomic profiles of analysed diatoms. In our experiments even the nitrogen-limited profiles of different isolates of the same species (*T*. *pseudonana* CCMP 1335 vs. *T*. *pseudonana* CCMP 1007; *T*. *weissflogii* CCMP1336 vs. *T*. *weissflogii* CCMP 1587) showed high similarity to the profiles of samples from control diatoms grown in nitrogen-replete conditions (Fig 2). This suggest an idea that nitrogen limitation may be changing the profiles (lipid and primary metabolites) within boundaries set by the genetic background of the species as displayed by the clustering of the low nitrogen profiles with respective controls.

Hu et al [30] reported an average lipid content of 22.7% dry weight for oleaginous diatoms grown under nitrogen-replete conditions, whereas the total lipid content under nutrient depletion stress reached 37.8% dry weight. The increase in total lipids of stressed diatoms is mainly explained by the shift in metabolism, which directs *de novo* synthesis and conversion of membrane polar lipids towards triacylglycerols. Many previous studies describe total changes in lipid content or abundance of specific classes in N-limited diatoms [22,30,32,33]. In our experiments we observed an accumulation of TAG (showing increase of specific TAG type levels) in low-nitrogen samples; however this trend was not consistent across analysed diatoms. This could be explained by the adaptation of cellular processes, which adapt carbon flux to the growth conditions such as mineral nutrient availability. The differences in accumulation of TAG under N-limitation could be explained by the different strategies of nitrogen utilization by these diatoms. For instance, the diverse response of diatoms on the molecular level might be explained by the assumption that the applied uniform nitrate concentrations (880 μM for high-nitrate control, 176 μM for low-nitrogen treatment) might fall into different specific growth optima. Further, these organisms differ in profiles of primary (especially nitrogen-containing) metabolites (Fig 4), which could be considered snapshots of metabolic steady states. Larger pools of soluble inorganic and organic N-containing compounds may be mitigating the N-limitation stress. It has been shown previously that diatoms can store nutrients in their vacuoles allowing several cell-divisions [34].Even though there was no detectable nitrate in the culture medium for two days, diatoms in our experiments could still be utilizing nitrate that was stored in vacuoles. Moreover, the availability of nitrogen remobilized from internal structures such as ribosomal proteins and photosynthesis-related proteins, might strongly differ as much as size of the analyzed diatoms. Taken together this provides a plausible explanation of discrepancies in TAG accumulation observed between species.

The regulation of the lipid biosynthesis pathway in diatoms cells under N stress has not yet been studied in detail [35]. Previous research on *T*. *pseudonana* and *Phaeodactylum tricornutum* in stressed by N-limitation showed reduced cell protein content, down-regulation of photosynthesis and a significant increase of glycolytic enzymes [36–38]. This, as well as the increased expression of fatty acid synthesis genes [37,38], suggest that increased amounts of acetyl-CoA coming from degradation of storage glucans may be feeding *de novo* synthesis of fatty acids and subsequently the accumulation of TAG in nitrogen limited diatoms. Moreover, a current hypotheses explain TAG accumulation under N-limitation by protein degradation and catabolism of amino acids (especially branched amino acids), which generates tricarboxylic acid cycle intermediates, such as malate, that are used for fatty acid biosynthesis [39]. The N-limitation has an impact on lipids not only through down-regulation and degradation of photosynthetic complexes but also affects structural lipids of plastids. Lipids of the thylakoid membranes such as MGDG, DGDG and sulfoquinovosyldiacylglycerol are central to photosynthetic function of a chloroplast involvement in stabilization of photosynthetic complexes [40,41]. Recent experiments on *Nitzschia closterium f*. *minutissima* [42] and *Phaeodactylum tricornutum* [38] reported N-limitation-induced mobilization of MGDG and DGDG with a consequent degradation of thylakoid membranes and increase in TAG [38].

Diatoms such as *B*. *biddulphiana* and *A*. *antediluviana*, which form large cells, displayed the highest amount of lipids per cell. In contrast, *T*. *weisflogii* and *T*. *pseudonana*, diatoms with relatively small cell size that grow to very high densities, appear to have low lipid content per cell. One possible explanation for this is the previously observed exponential relationship between amount of lipid per cell and cell volume [43].

The most abundant group within TAG are lipids with 48 to 54 C in acyl chains (with different desaturation grades). They are most likely composed of glycerol bound with three of the main cellular fatty acids: palmitic (FA16:0) and stearic (FA18:0) acids and their desaturated derivatives. The abundance of TAG48:0 is in agreement with data obtained previously with approaches focusing on the fatty acids in diatom lipids [44]. These previous studies showed a high proportion of FA16:0 in cellular fatty acids of diatoms. Thus, one can assume that TAG48:0 is comprised of glycerol and three palmitic acid moieties. Some diatoms can accumulate more than 15% of their total fatty acids in form of myristic acid (FA14:0) [44–46]. Myristic acid has been shown to be incorporated into positions *sn–1* and *sn–3* in TAG from *T*. *pseudonana* [47]. Unfortunately, given the fact of high abundance of 14 C, 16 C, and 18 C fatty acids, the acyl chains of most of the lipid species cannot be unambiguously deduced without special analytical approaches. Recently, a fatty acid profiling revealed that in cells of *T*. *pseudonana* eicosapentaenoic acid (FA20:5) and docosahexaenoic acid (FA22:6) are the most abundant fatty acids [48]. On the other hand, the cells of *B*. *biddulphiana* accumulated much less of these fatty acids. The most intense peaks in fatty acid profiles of this diatom correspond to arachidonic acid (FA20:4), while content of the other poly-unsaturated FA (FA20:5 and FA22:6) was significantly smaller [48]. Moreover, the level of FA18:4 in *B*. *biddulphiana* is much higher when compared to *T*. *pseudonana*. Such differences in fatty acid profiles have been already reported by Dunstan et al. [49]. In his collection of studied algae, diatoms described as Centrales accumulated FA18:4, while Pennales, were relatively rich in FA20:4. Additionally, significantly higher proportion of FA22:6 was observed in fatty acid profiles of Centrales than of Pennales [49]. One should note that the evolutionary history of diatoms is more nuanced than the centric (Centrales)/pennate (Pennales) division. According to recent diatom taxonomy both *Thalassiosira* and *Biddulphia* are Mediophyceaen [50,51]. *Thalassiosira* and *Biddulphia* do differ in habitats—*Thalassiosira* is small-celled planktonic diatom while *Biddulphia* forms colonies in benthic/epiphytic habitats. These aspects should be taken into account while interpreting the observed molecular phenotype of those diatoms.

In plants galactolipids can be synthesised in two pathways: one “prokaryotic” and the other “eukaryotic”. The main difference between the pathways, other than their subcellular localization, is the enzyme preference for fatty acids at the *sn*–1 and *sn*–2 positions of glycerolipids. [52]. The “prokaryotic” pathway is localized to the chloroplast and the MGDG and DGDG synthesised there contain predominantly 18-C fatty acids in the *sn*–1 position and 16-C fatty acids in the *sn*–2 position. In the “eukaryotic” pathway, which is localized to the endoplasmic reticulum, FA18:1 (oleic acid) is incorporated into phospholipids in the *sn–2* position. These phospholipids are the source of DAG for chloroplast-localized synthesis of galactolipids. Hence galactolipids synthesized according to the “eukaryotic” pathway contain predominantly 18-C fatty acid in both *sn*–1 and *sn*–2 positions [52]. Mono- and digalactosyldiacylglycerols from diatoms are mainly synthesised in the “prokaryotic” pathway, as these compounds contain 16-C fatty acids in the *sn–2* position [53,54]. Thus, we can deduce the fatty acid chain length in galactolipids presented in this report. Five out of nine MGDG detected in analysed diatoms are probably MGDG 18-C/16-C. In the case of MGDG 36:6, we speculate that arachidonic acid (FA20:4) or eicosapentaenoic acid occurs in the *sn–1* position. The plant model of galactosylglycerols synthesis is most likely true for diatoms as well, which is supported the study of Dodson et al. [53] who showed that galactolipids from *T*. *pseudonana*, *Skeletonema marinoi* and *P*. *tricornutum* contain primarily 20:5/16-C fatty acids. Similar conclusions about the fatty acid composition can be made for DGDG 36:6. Both galactolipids are the most abundant compounds in their classes in all 13 analysed cultures of diatoms. Radiolabeling experiments showed that in *P*. *tricornutum* FA20:5 is synthesized in microsomes [55]. The authors hypothesized that the FA20:5 can be transported to plastids as a free fatty acid or bound in DAG [55]. Regardless of whether the fatty acids are derived from lipid catabolism or *de novo* biosynthesis, diacylglycerol acyltransferases catalyze DAG conversion into TAG [56]. In our data, out of ten molecular species of the DAG class only DAG36:6 is found in all diatom extracts. The intensity and widespread detection of this compound suggest its high importance in the diatom lipid metabolism.

### Primary metabolites profiling

In recent years there have been several publications presenting metabolic profiles of diatoms, including *T*. *pseudonana* [7], *P*. *tricornutum* [57], *Skeletonema marinoi* [58] and *Cocconeis scutellum* [59]. However, our knowledge of metabolism of these organisms is still limited. To increase the knowledge in this field this paper presents metabolic profiles for 13 different species and strains of marine diatoms cultivated in N-replete and N-limiting conditions.

The central metabolism of the studied diatoms is also affected by the applied growth conditions with reduced nitrogen level, although the type of response was very different between species (Fig 4). For example, *H*. *sinensis* and *A*. *antediluviana* have reduced levels of most of measured compounds, whereas the three *Thalassiosira* species tend to accumulate various compounds as adaptation mechanisms to low-nitrogen conditions. This contradicts previously published metabolic profiles of *T*. *pseudonana* from cultures that were transferred from N-replete to nitrate-free medium [7]. In this report, primary amino acids levels, as main N-containing primary metabolites, where strongly reduced. An additional characteristic change in response to the N-limitation is the accumulation of citrate. This was observed in the short term medium shift experiments with *T*. *pseudonana* [7] and in nitrogen limitation experiments on *Chlamydomonas reinhardtii* [60]. Moreover, previous proteomic experiments on *T*. *pseudonana* have shown an increase of glycolytic and TCA enzymes in N-limited cells [36]. Increased levels of citrate were observed in *H*. *sinensis*, *B*. *biddulphiana*, and *T*. *pseudonana* CCMP1335 but only the profile of *Ch*. *simplex* showed a strong increase of citric acid in N-limited cells in comparison to the N-replete control. In our experimental conditions *Ch*. *simpex* samples grew very rapidly, which may have resulted in very high demand for N and at the time of harvest strong accumulation of TCA intermediates, including citrate. This could explain the difference between *Ch*. *simpex* and other slower growing diatom cultures, which could better adapt to the limited availability of nitrate in the media (e.g. slowed down amino acid synthesis) and consequently the over-accumulation of photosynthetic carbon.

## Conclusions

We have extracted and analysed lipids from 13 different strains and cultures of diatoms in order to explore the diversity of lipid metabolites in these organisms grown under two different conditions, i.e. nitrogen replete and nitrogen deplete conditions. The diatoms analysed represent different species and genera, shape and size, life habitats (benthic and planktonic) and colonization types (free living and chain forming). We identified 142 annotated lipid species mostly from the TAG class, many of them displaying species specificity. The growth in nitrogen-deplete or nitrogen-replete conditions affects lipid accumulation but has no major influence on the lipid profiles. We also observed an increase of TAG accumulation in low nitrogen samples, although this trend was not consistent across all 13 diatom strains. Overall, our results confirm that diatoms display a large diversity of lipid species. This observation is consistent with previous suggestions regarding the value of diatoms for producing lipids for either bioenergy or as feed stock.

## Supporting Information

S1 Table Normalized and log2-transformed intensity data of 142 annotated analytes measured in extract of 13 diatom strains. Following culture abbreviations were used: Ampant, *Amphitetras antediluviana*; Bidbid, *Biddulphia biddulphiana*; Cerdea, *Cerataulina daemon*; Chasim, *Chaetoceros simplex*; Eunlae, *Eunotogramma sp*.; Hemsin, *Hemiaulus sinensis*; Lepdan, *Leptocylindrus danicus*; Rhiset, *Rhizosolenia setigera*; Thafra, *Thalassionema frauenfeldii*; Tp1007, *Thalassiosira pseudonana* CCMP1007; Tp1335, *Thalassiosira pseudonana* CCMP 1335; Tw1587, *Thalassiosira weissflogii* CCMP 1587; Tweiss1336, *Thalassiosira weissflogii*. The “N” suffix marks samples from low nitrogen growth conditions.
(XLS)

S2 Table Log2-transformed ratios of 96 primary metabolites and corresponding T-test p-values. Following culture abbreviations were used: Ampant, *Amphitetras antediluviana*; Bidbid, *Biddulphia biddulphiana*; Cerdea, *Cerataulina daemon*; Chasim, *Chaetoceros simplex*; Eunlae, *Eunotogramma sp*.; Hemsin, *Hemiaulus sinensis*; Lepdan, *Leptocylindrus danicus*; Rhiset, *Rhizosolenia setigera*; Thafra, *Thalassionema frauenfeldii*; Tp1007, *Thalassiosira pseudonana* CCMP1007; Tp1335, *Thalassiosira pseudonana* CCMP 1335; Tw1587, *Thalassiosira weissflogii* CCMP 1587; Tweiss1336, *Thalassiosira weissflogii*.
(XLS)
